# Mechanisms for cell survival during abiotic stress: focusing on plasma membrane

**DOI:** 10.1007/s44154-024-00195-5

**Published:** 2025-01-02

**Authors:** Xiao Su, Lijuan Yao, Xuechen Wang, Yuan Zhang, Guifang Zhang, Xiaojuan Li

**Affiliations:** 1 State Key Laboratory of Efficient Production of Forest Resources, College of Biological Sciences and Technology, Beijing Forestry University, Beijing, 100083 China; 2 Key Laboratory of Genetics and Breeding in Forest Trees and Ornamental Plants, Ministry of Education, College of Biological Sciences and Technology, Beijing Forestry University, Beijing, 100083 China

**Keywords:** Abiotic stress, Plasma membrane integrity, Signal transduction, Stress tolerance

## Abstract

Plants are continually challenged by abiotic stressors, including drought, salinity, and extreme temperatures, which can adversely affect their growth and development. The plasma membrane, acting as a pivotal interface between the cell and its environment, is particularly susceptible to such stresses. This review focuses on current understanding of how abiotic stresses affect plasma membrane integrity in plants. The review also explores the critical roles of plasma membrane proteins and lipids under stress conditions, highlighting signal transduction pathways that the plasma membrane initiates to mitigate abiotic stress. By consolidating these findings, this review provides a comprehensive overview that is pivotal for advancing the development of stress-tolerant plant varieties. The insights gained from this synthesis are expected to contribute significantly to the enhancement of plant resilience in the face of environmental challenges.

## Introduction

The growth, development, and geographic distribution of plants, as well as the quality and yield of crops, are profoundly influenced by their growing environment. Among various environmental factors, abiotic stress significantly affects agricultural productivity, plant growth and development (Li et al. 2019; Miyazaki et al. 2021; Pei et al. 2022; Islam et al. 2024; Liang et al. 2024). Adverse abiotic conditions, such as high temperature, low temperature, drought, and salt, pose substantial challenges to plant health (Zhang et al. 2020; Li et al. 2022a, b; Kopecká et al. 2023; Wang et al. 2024). Plants have evolved various defense mechanisms enabling them to adapt abiotic stress at both physiologically and molecularly level (Li et al. 2022a, b; Kopecká et al. 2023; Kuppusamy et al. 2023; Rui and Wang.^2024^; Rahman et al. 2024).

The plasma membrane (PM) serves as a crucial barrier between the cell and its surroundings, playing a pivotal role in maintaining intracellular stability and facilitating substance exchange with the external environment (Medina-Puche and Lozano-Durán. 2022). Meanwhile, the PM, which comprised predominantly of membrane proteins and lipids, acts as the primary environmental sensor in plants (Sun et al. 2022). Environmental signals are primarily detected and transduced through changes in membrane lipids and membrane proteins. Furthermore, the PM exhibits fluidity, selectivity, and other characteristics essential for the normal cellular activities. Therefore, maintaining the integrity of the PM is crucial for the proper functioning of plant PM.

Under abiotic stress conditions, the PM is highly susceptible to damage. Drought-induced osmotic stress affects the structural integrity of the PM, altering its permeability and thereby increasing the pressure on plants to survive in water-deficient environments (Munnik et al. 2021; Ozturk et al. 2021). Salt stress also induces osmotic stress on the PM. Additionally, salt stress introduces ion injury to the PM, and the combined effects of osmotic stress and ion injury slowly destruction the structural integrity of the PM, further intensifying the survival challenges for plants in high-salinity environments (Arif et al. 2020; Zhou et al. 2024). Low temperatures stress causes physiological drought by inhibiting plant water uptake and transport, leading to drought-like symptoms despite the presence of soil moisture. Low temperatures stress also impairs plant growth and development, causing strength loss and surface lesions, as well as biochemical changes at the enzymatic level alter the viscosity, permeability, and fluidity of PM (Almadanim et al. 2018; Ruiz-Lopez et al. 2021). High temperatures also present significant challenges to the integrity of PM and should not be disregarded, which are exacerbated by the heightened fluidity of membrane lipids, the process of lipid peroxidation, and the degradation of proteins across various metabolic pathways. (Burgos et al. 2011; Bourgine and Guihur 2021; Ruiz-Lopez et al. 2021; Zhao et al. 2021a, b; Sharma et al. 2023).

To withstand environmental stresses, plants have developed intricated regulatory networks that allow them to react and adjust to their surroundings. This involves the stress adaptation model of plants under abiotic stress (Gusain et al. 2023). In this model, the PM is regarded as a dynamic system, which does not passively endure stress but can sense stress signals (Nguyen et al. 2018). In this review, we summarize recent researches on the damages on the PM under abiotic stress and the mechanism of PM response to abiotic stress, providing references for future research on plant responses to abiotic stress.

### Plasma membrane integrity is important for plant

The PM is not only a basic physical barrier shielding the cell interior from the external environment, but also a selective barrier that facilitates a multitude of precisely regulated cell functions, including signal transduction, cellular communication (Amorim-Silva et al. 2019; Jaillais and Ott 2019; Fichman et al. 2022). Composed of lipids and proteins, PM is equipped with a variety of receptors and sensors that can recognize changes in factors such as temperature, humidity, and the presence of various molecules (Jiang et al. 2019; Zhao et al. 2019). Upon sensing these environmental cues, the PM initiates a cascade of events by converting the perceived changes into specific signals. These signals are then relayed through a network of signaling pathways within the cell. These pathways involve the activation of enzymes, generation of second messengers, and modulation of gene expression, ultimately leading to a coordinated cellular response that aligns with the new environmental conditions. This intricate process ensures that the plant can adapt and survive in a dynamic and ever-changing world (Zandalinas and Mittler 2021).

Proteins at the cell surface, which use the PM as a platform, are essential for a variety of cellular functions and biological processes (Gouguet et al. 2021). As one of the most important organelles in the cell, the PM is responsible for a variety of physiological processes that rely on its structural integrity. When PM integrity is compromised, transmembrane transport may also be impacted. For example, obstacles to material transport will affect the absorption of nutrients and the discharge of waste by plant cells, thus interfering with metabolic processes (Li et al. 2023). In the long term, damage to the PM integrity lead to electrolyte leakage even cell death, results in dwarfing, shrinkage of the leaves, as well as reduced biomass yield (Kim et al. 2016; Petrov et al. 2018; Medina-Puche et al. 2020; Ruiz-Lopez et al. 2021; Kopecká et al. 2023; Baena et al. 2024). Therefore, in order to enhance the capacity to environmental changes, it is imperative that the integrity of the PM be preserved and repaired.

### The damage to plasma membrane under the different abiotic stresses

Growing evidence indicate that stress-induced damages on integrity of membrane systems, are closely associated with disrupting cellular homeostasis and function (Yamazaki et al. 2008; Li and Kim 2022). In this section, we provide a detailed summary of the damages inflicted on the PM by various stress factors, evaluating the physiological and biochemical impacts on plant health and resilience.

### Low temperatures stress

Plant growth, development, distribution, and seasonal behavior are all significantly influenced by temperature. Both excessive low and high temperatures are detrimental to the of plants (Adhikari et al. 2021; Ding et al. 2020). Freezing injury, occurring below 0 °C and cold stress, encompassing temperatures ranging from 0 to 20 °C, are common abiotic stresses to plant cells. These stresses impact the PM through distinct mechanisms. Freezing injury is characterized by the formation of ice crystals, which lead to direct mechanical damage and cell dehydration. This ultimately results in structural alterations in the PM, increased electrolyte leakage, and overall membrane damage (Schapire et al. 2008; Yamazaki et al. 2008). Whereas, cold stress primarily affects membrane fluidity and the functionality of membrane proteins, without the formation of ice crystals (Sangwan et al. 2002). Research has shown that the lipid content of PM can alter in response to cold stress. When plants are exposed to cold stress, PM retains an excessive amount of diacylglycerol, reducing fluidity of PM and then cause rupture of PM (Ruiz-Lopez et al. 2021). Low temperature was also reported to reduce PM fluidity of pineapple. The integrity of PM lost, according to the ultrastructure observation of pineapple cells undergo low-temperature storage (Zhang et al. 2011). Moreover, phosphatidylinositol (PI) levels dropped, PM ATPase activity decreased, and PM phosphatidylic acid (PA) levels rose (Zhou et al. 2014). It is postulated that low temperature may affect PM ATPase activity and decrease PM integrity by changing the lipid composition of the PM, based on the effect of PA level on PM ATPase activity(Zhou et al. 2014; Ponce-Pineda et al. 2021). Both freezing and cold stress can lead to severe PM damage, including expansion-induced lysis (EIL) and loss of osmotic responsiveness (LOR), underscoring the critical need to maintain PM integrity under such environmental challenges (Uemura et al. 2003).

### High temperature stress

High temperatures have emerged as a primary challenge in global crop production (Larkindale and Vierling 2008; Saidi et al. 2009; Zhai et al. 2017; Djanaguiraman et al. 2018a, b; Bheemanahalli et al. 2019; Haider et al. 2021). Transmission electron microscopy (TEM) analysis of wheat cells ultrastructure showed damage to the PM under high temperature stress (Djanaguiraman et al. 2018a, b). Although the exact cause of this damage remains unclear, the research indicated that the PM was negatively impacted by the high temperatures. Lipid peroxidation, protein denaturation, and imbalance of membrane fluidity may all induce PM damage. Plant peroxidase activity increases in response to high temperature stress, damaging membrane lipids and associated proteins (Li and Kim 2022). Lipid peroxidation within PM is often gauged by the level of malondialdehyde (MDA), a substance that poses a threat to cellular health when its concentration increases (Hu et al. 2023). Moreover, elevated membrane fluidity, resulting from increased lipid unsaturation levels and stigmasterol content, can lead to PM rupture. All these factors collectively contribute to the observed PM damage (Djanaguiraman et al. 2018a, b; Raja et al. 2020; Hu et al. 2023).

### Drought stress

Drought stress significantly reduces the relative water content and transpiration rate in plants, leading to the oxidation of unsaturated fatty acids and protein degradation. It also damages the semipermeability and integrity of the PM, resulting in cytosolic electrolyte leakage (Kocheva et al. 2009; Petrov et al. 2018). The reduction of turgor pressure and the alteration in plant cell PM tension caused by excessive osmotic stress could be the source of the damage to PM induced by drought (Kocheva et al. 2009, 2014; Shi et al. 2023; Guo et al. 2024; Zhang et al. 2024). Under drought conditions, the phospholipid degradation in winter wheat leaves were primarily phosphatidylcholine (PC), phosphatidylethanolamine (PE), and phosphatidylglycerol (PG), with PC experiencing the greatest degradation. Membrane electrolyte leakage significantly increased after these phospholipids decreased, indicating that severe phospholipid degradation precedes membrane integrity loss. Degradation of PM phospholipids may contribute to the loss of the PM integrity (Wang et al. 2020). In addition, drought can induce oxidative stress in plants (Fu et al. 2023). Drought-induced oxidative stress damages plant membrane systems through ROS. Plants have developed defense mechanisms against oxidative stress and plant drought stress tolerance is directly correlated with the effectiveness of their enzymatic scavenging mechanisms. Suppressing the expression of ldi-miR396b upregulated LdPMaT1 during drought stress in lily, enhancing ROS scavenge capacity and drought resilience (Fu et al. 2023). Collectively, drought stress damages the plant PM through hypertonic osmotic stress, lipid oxidation, and phospholipid degradation, as well as oxidative stress. Plants counter these challenges using enzymatic scavenging mechanisms localized at the PM.

### Salt stress

Soil salinity, characterized by the predominance of soluble ions in the soil, negatively impacts on photosynthetic capacity, transpiration rate, enzymatic activities, cellular homeostasis, metabolism, ion transport, and plant morphology (van Zelm et al. 2020; Zhao et al. 2021a, b; He et al. 2022; Joshi et al. 2022; Liu et al. 2022; Morita et al. 2023; Xiao and Zhou 2023; Zhou et al. 2024). For example, salt stress led to the synthesis and accumulation of ROS, which interferes activity of enzyme localized on PM and alters the membrane permeability (Arif et al. 2020; Joshi et al. 2022; Ma et al. 2023).

de Freitas et al. reported that salt stress damages the PM of sorghum cells within seven days, leading to electrolyte leakage (de Freitas et al. 2019). Furthermore, research in rice also shows that salt stress can cause electrolyte leakage in plant leaves, indicating PM damage in rice leaf cells under salt tolerance (Roy et al. 2022). In summary, salt stress can damage the PM through various mechanisms, including osmotic stress, ion toxicity, ROS outbreaks, and membrane protein denaturation (Guo et al. 2019; Lv et al. 2021).

### Function of plasma membrane in response to stresses

In response to the challenge of abiotic stress, the PM has evolved complex mechanisms to enhance stress tolerance and maintain cellular integrity. Lipid composition and contents maybe remodeled, and numerous PM proteins (Table 1) functioned as sensors to transmit precise signals to the cell’s interior (Manasa et al. 2022; Ramazam et al. 2023).

**Table 1:** Membrane proteins involved in abiotic stress response

Proteins	Category	Types involved in abiotic stress responses	Description	References
CRLK1	Kinases and phosphatases	Low temperature	Activating the MAPKs	(Yang et al. 2010)
CRPK1		Low temperature	Phosphorylating target proteins to enhance cold tolerance	(Liu et al. 2017)
COLD1	Receptor proteins	Low temperature	Sensing the cold signal and interacting with RGA1 to enhance Ca^2+^ influx	(Ma et al. 2015)
SOS1	Transporter proteins	High salt	Maintaining ion balance and reducing Na ^+^ toxicity	(Lu et al. 2023)
MCAs	Ion channels proteins	Low temperature Drought	Promoting Ca^2+^ influx	(Yoshimura et al. 2021)
AKT1		Salt	Facilitating K ^+^ uptake and osmotic adjustment	(Li et al. 2023)
MSL10		Drought	Sensing osmotic stress and enhance Ca^2+^ influx	(Basu et al. 2020)
OSCA1.2		Drought	Enhancing to osmotic adjustment and drought resistance by regulating Ca^2+^ influx	(Han et al. 2024)
CNGCs		High temperature	Promoting Ca^2+^ influx	(Niu et al. 2020)
RbohD	Hydrolases	High temperature	Elevating ROS to activate the MAPKs	(Rivas et al. 2024)
PIP2	Aquaporins	Salt	Regulating osmotic water flow	(Baena et al. 2024)

### Plasma membrane lipid remodeling in stress resistance

Lipid diversity and homeostasis are important for PM to respond against external pressures. By increasing the synthesis of specific lipids or reducing their degradation, plants adjust the fluidity and phase transition temperature of the membrane to adapt to varying environmental conditions (Bakht et al. 2006; Tarazona et al. 2015; Tshabuse et al. 2018; Guo et al. 2019; Sharma et al. 2023). For instance, in winter wheat leaves under drought conditions, the contents of PC, PE, and PG in the PMs exhibited a very similar trends relative to stress duration. Membrane lipid fluidity is positively correlated with Double Bond Index (DBI), which serves as an important metric for assessing membrane integrity. After 8 h of drought stress, the DBI of PC increased significantly and persisted for several days. Whereas, the DBI of PI, PG, and Phosphatidylserine (PS) significantly decreased at different times within the first 1–4 days after drought stress, while no notable alteration was observed in the DBI of PE. These findings indicated that PC was the primary phospholipid that responds to water stress by increasing unsaturation (Wang et al. 2020). Changes of the composition and degree of desaturation of fatty acids were also detected under drought stress. It was reported that drought stress led to a significant decrease in the contents of C16:1, C16:3 and C18:3 fatty acids and an increase in the levels of C16:0 and C18:2 fatty acids (Gigon et al. 2004; Liu et al.^2019^; Yin et al. 2024).

A thorough examination of lipidomics and transcriptomics revealed alterations in lipid metabolism in the root membrane of maize seedlings under low temperature stress. Cold stress from low temperatures caused an increase in phosphatidic acid (PA) and PE levels, alongside a decrease in PC content. A reduction in PC levels can compromise membrane integrity under stress, while an increase in PA content helps to mitigate cold stress damage in maize seedlings (Zhao et al. 2021, b). Another research demonstrated the significance of PC in plant stress resistance by showing that it serves as the primary substrate for the activation of phospholipase Dα (PLDα) induced by freezing. PLDα activity might contribute to freeze-induced lipid hydrolysis and damage the integrity of the PM. During freezing, increased lipolysis activity of PLDα altered the composition of membrane lipids, with the wild PC of type level decreasing significantly more than those in PLDα-deficient plants (*pldα*). This corresponds with increased cold tolerance in *pldα* (Welti et al. 2002). Diacylglycerol (DAG) was also detected to be large accumulate in plant PMs under low temperature stress, which reduces the fluidity of membrane and increased the risk of membrane rupture. To preserve the PM integrity and fluidity, the PM protein SYT1 can bind DAG on the membrane and transfer it to the ER using its special SMP structure (Ruiz-Lopez et al. 2021; Qian et al. 2022).

In response to heat stress, heat-tolerant soybean genotype DS25-1 showed significantly lower expression levels of FAD3A and FAD3B genes compared to heat-susceptible genotype DT97-4290. This led to a decrease in the content of polyunsaturated linolenic acid (18:3), which consequently led to a decrease in the level of lipid unsaturated fats. The decrease in18:3 in DS25-1 aids in the preservation membrane function and high-temperature tolerance (Narayanan et al. 2020). A similar adaptation mechanism to heat stress in peanuts is reported by lipidomic analysis in peanut (*Arachis hypogaea L.*) anthers. Under heat stress, lipid unsaturation levels according to the amount of 18:3 fatty acid was reduced through downregulate FAD3 expression. (Zoong Lwe ZS et al. 2020). In TrFQR1-transgenic white clover (*Trifolium repens*), the heat resistance is enhanced through an increase in lipid saturation and the ratio of PC to PE, which improve PM integrity and stability under heat stress conditions (Cheng et al. 2023).

Lipid remodeling in response to salt stress varies among different plant species (Yu et al. 2021). For instance, in the halophyte ice plant (*Mesembryanthemum crystallinum* L.), an increase in non-bilayer forming and negatively charged lipids (PE and PS) was observed in the PM under salt stress (Guo et al. 2019, 2022). Positive correlations between the PS level and plant salt tolerance were also reported in *Salicornia europaea* L. The increased PS level minimizes membrane damage, reduces PM depolarization, and maintains K^+^/Na^+^ balance, thus enhances salt tolerance of the transgenic lines (Lv et al. 2021). In Barley (*Hordeum vulgare*), PCs play crucial roles in the concentration-dependent adaptive reactions to salt stress(Sarabia et al. 2020). Growing number of highly unsaturated PC species improved membrane fluidity, which may protect PM integrity and enhance resistance to salt stress.(Sarabia et al. 2020).

Plants respond to stressors partly through changes in lipid content. The stability of plant membrane lipids contribute to stress resistance, and lipids also participate in the transduction of abiotic stress signals as signaling molecules. Thus, exploring the physiological role of plant lipids under stress has significantly theoretical and practical implications.

### Key roles of membrane-localized proteins in perception and response to abiotic stress

Plants constantly sense changes of the environment and integrate this information to develop a coordinated, whole-plant response strategy. This adaptive capability is enabled by a complex network of molecular, chemical, and physical elements that collaborate within a sophisticated communication framework (Gusain et al. 2023; Xiao and Zhou 2023; Ruan et al. 2024). To mitigate the effects of abiotic stressors and ensure survival under adverse conditions, plants have evolved a plethora of molecular mechanisms designed for detection, response, and adaptation to unfavorable environmental scenarios.

**Fig. 1:**
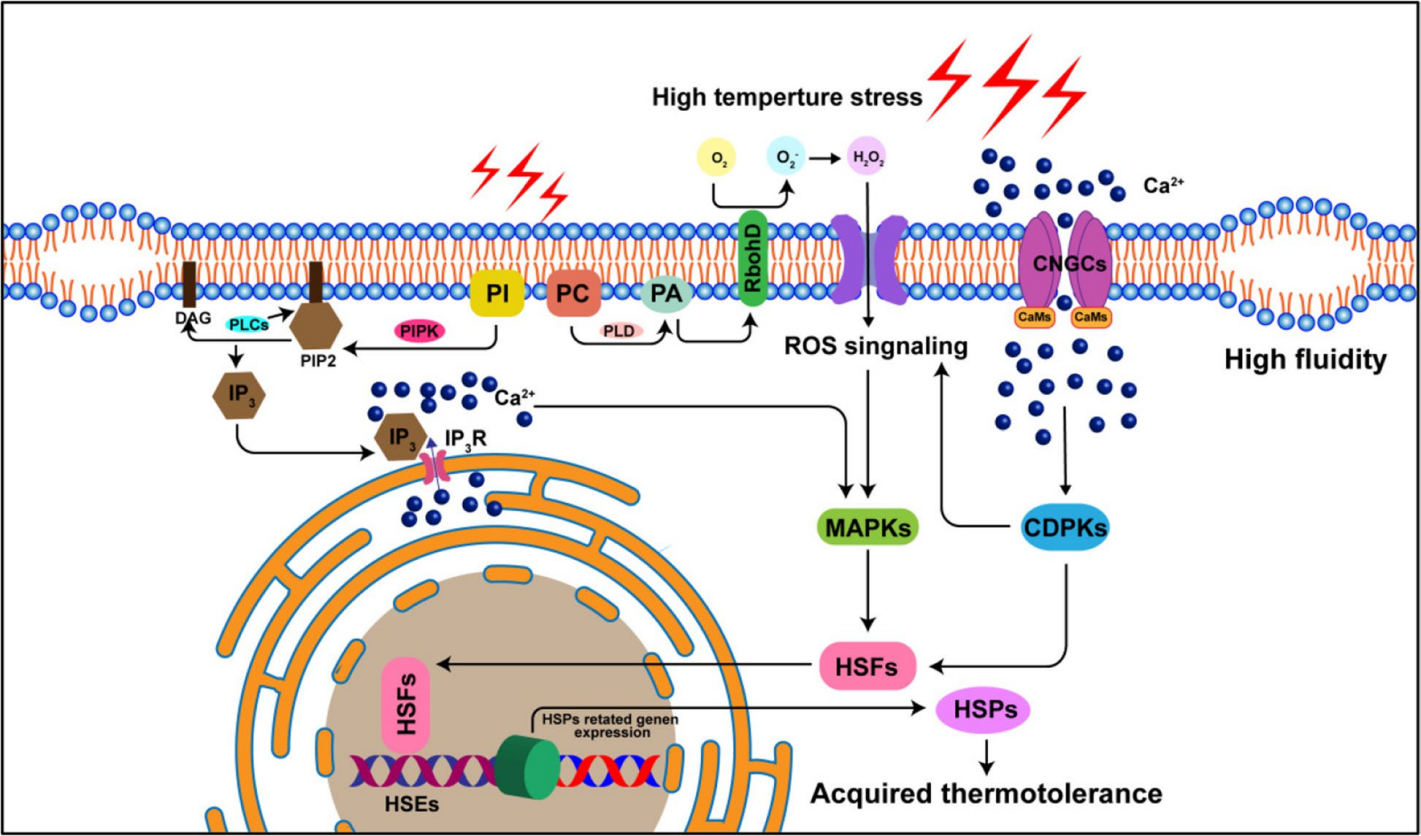
Diagram illustrating the signal transduction pathways that high temperture stress activates. Heat stress affects membrane fluidity and stability, causing Ca^2+^ to enter the cytosol through the opening of cyclic nucleotide-gated channels (CNGCs) on the PM. This influx of Ca^2+^ triggers a specific signaling cascade where Ca^2+^ binding to CNGC-bound CaMs activates kinases. These kinases then phosphorylate and activate heat shock transcription factors (HSFs). Additionally, membrane instability can initiate lipid signaling through phospholipase D (PLD) and phosphatidylinositol phosphate kinase (PIPK) enzymes. Phospholipase C (PLC) hydrolyzes phosphatidylinositol 4,5-bisphosphate (PIP2) to produce inositol trisphosphate (IP3), which interacts with IP3 receptors (IP3R) to release intracellular Ca^2+^ from the endoplasmic reticulum (ER), further initiating a signaling cascade. Cytosolic Ca^2+^ binds to calcium-dependent protein kinases (CDPKs) and CaM3, activating HSFs in the cytosol and enhancing reactive oxygen species (ROS) production through the phosphorylation of NADPH oxidase, specifically the RBOHD. Eventually, HSFs translocate into the nucleus and bind to heat shock elements (HSEs) to promote the expression of heat shock protein (HSP) related genes

PM plays a crucial role in heat sensing, containing calcium channels, such as cyclic nucleotide-gated channels (CNGCs), that respond to temperature changes by mediating Ca^2+^ entry and triggering heat-shock proteins (HSPs) accumulation (Fig. 1) (Bourgine and Guihur 2021; Guihur et al. 2022). Moreover, during heat stress, the Phospholipase C Isoform 3 (PLC3) and PLC9, which take phosphoinositides (PI) as substrate, are quickly activated in the initial stage of a heat stress, leading to cytosolic Ca^2+^ accumulates. The hydrolysis of phosphatidylinositol 4,5-bisphosphate by PLC3 and PLC9 results in the formation of DAG and inositol-1,4,5-trisphosphate (IP_3_), respectively. This process activates the release of Ca^2+^ from intracellular stores, triggering a signaling cascade involving kinases and calmodulins (CaMs) (Fig. 1) (Gao et al. 2014; Ren et al. 2017; Abdelrahman et al. 2020; Kumar et al. 2020). Subsequently, heat shock transcription factors are activated to enhance plant heat tolerance. (Ul Haq et al. 2019).

**Fig. 2:**
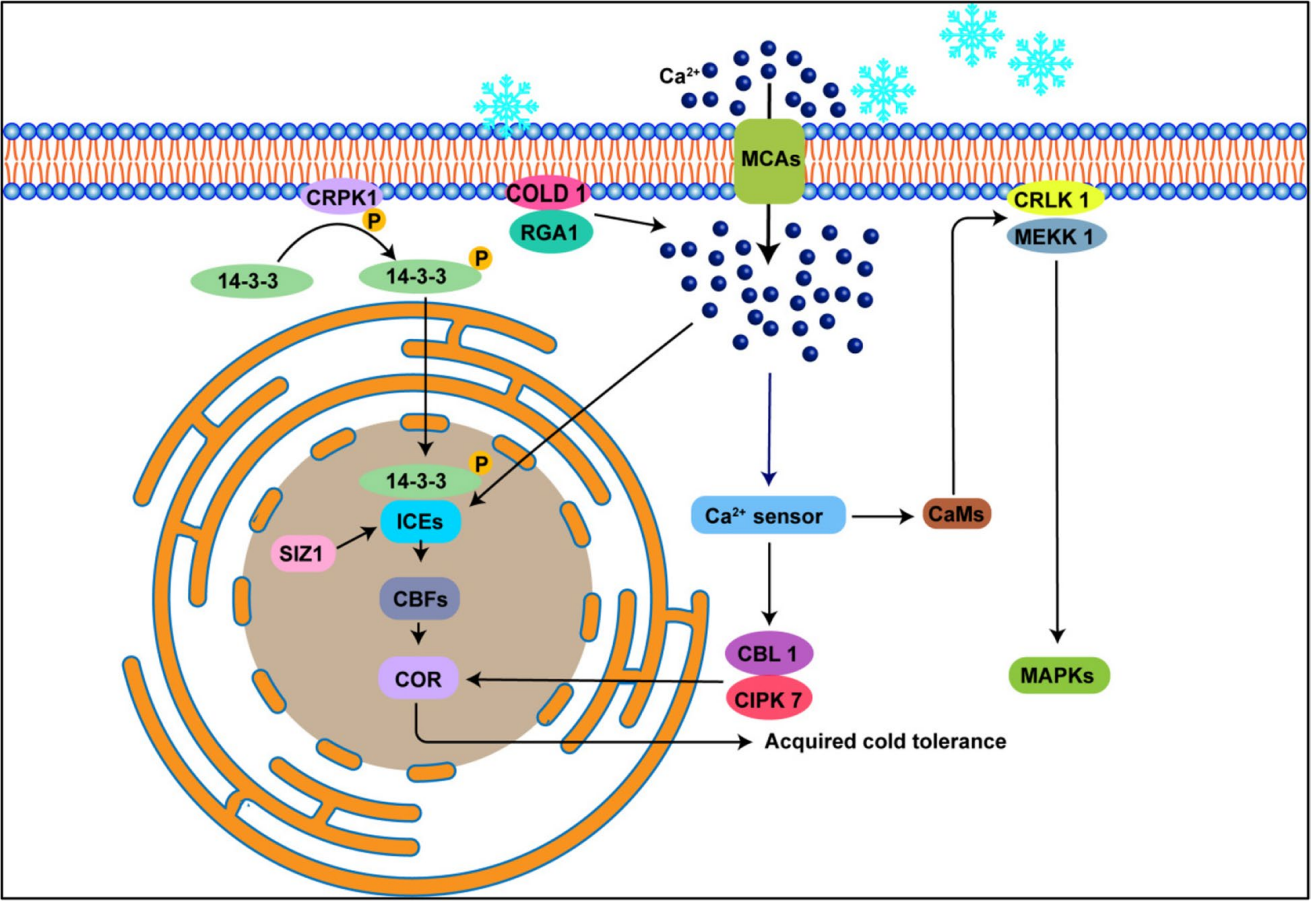
Diagram illustrating the signal transduction pathways that low temperture activates. During cold stress, the PM hardens and Ca^2+^ enters through mechanosensitive channels (MCAs). COLD1 detects the cold signal and interacts with RGA1 to enhance Ca^2+^ flow. To transmit cold signals, Ca^2+^/calmodulin (CaM) upregulates the kinase activity of calcium/calmodulin-regulated receptor-like kinase 1 (CRLK1) and facilitates its interaction with mitogen-activated protein kinase kinase 1 (MEKK1), activating the MAPKs. Calcium levels during cold stress are regulated by calcineurin B-like proteins through the serine-threonine protein kinase CIPK. In Arabidopsis, the upregulation of CBL1 and its interaction with CIPK7 create a freezing-tolerant phenotype and induce the expression of various Cold-Regulated (COR) genes. CRPK1 phosphorylates the 14–3-3 protein in the nucleus, facilitating the regulation of CBF-dependent cold signaling. The SIZ1-dependent sumoylation of the Inducer of CBF Expression 1 (ICE1) may activate and stabilize the protein, promoting CBF expression. The induction of CBF genes leads to the activation of C-repeat Binding Factors (CBFs), which target COR genes, contributing to increased freezing tolerance

In response to low-temperature stress, the PM serves as the primary sensor, rigidifying and decreasing in fluidity (Wei et al. 2021). The CHILLINGTOLERANCE DIVERGENCE 1 (COLD1) protein interacts with G-protein α subunit 1 (RGA1) to facilitate Ca^2+^ influx after sensing the cold signal (Ma et al. 2015). Ca^2+^ flowes by the activated Ca^2+^-permeable mechanosensitive channel (MCAs) (Gusain et al. 2023; Mori et al. 2018). These Ca^2+^ signals, detected by variety of Ca^2+^ sensors like CaMs and calcineurin B-like (CBLs), trigger the ICE-CBF-COR signaling pathway via the MAPKs cascade reaction, preserving PM integrity and enhancing cold tolerance ultimately (Fig. 2) (Adhikari et al. 2022; Hwarari et al. 2022; Gusain et al. 2023; Khan et al. 2023). Moreover, cold-activated PM protein CRPK1 modulates the CBF-dependent cold signaling by phosphorylating 14–3-3 proteins, promoting CBF proteins destabilization in the nucleus (Fig. 2) (Liu et al. 2017).

**Fig. 3:**
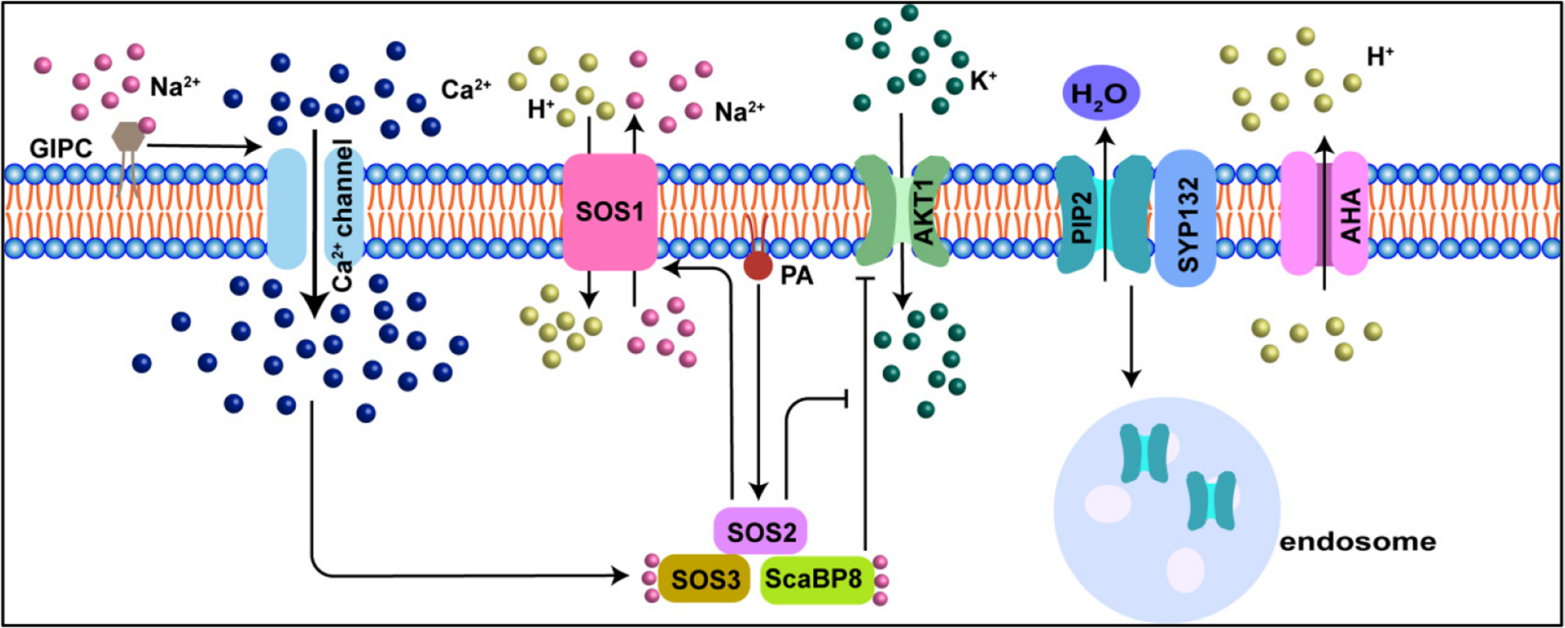
Diagram illustrating the signal transduction pathways that high salt stress activates. The SOS pathway plays a critical role in maintaining ionic homeostasis and may interact with other signaling networks to regulate plant salt tolerance more precisely. During salt stress, glycosyl inositol phosphoryl ceramide (GIPC), a type of negatively charged membrane lipid, likely contributes to salt sensing by directly binding to Na^+^. This interaction activates a calcium channel, leading to a Ca^2+^ influx that triggers an adaptive reaction to increased salt concentrations. The Ca^2+^-binding protein SOS3 activates the protein SOS2, which subsequently activates SOS1 to expel Na^+^ from the cell. For a precise plant salt stress response, intracellular calcium signals are effectively detected by Ca^2+^ sensors like SCaBP8. SCaBP8 interacts with SOS2 to inhibit the transport of K^+^ through AKT1 channels. Salinity reduces the amount of AHA1 binding to the SNARE complex but increases PIP2 binding to SYP132, facilitating the redistribution of PIP2 from the PM to endosomal membranes

When exposed to salt stress, plants rapidly detect excess Na^+^, triggering a sodium stress response cascade. Glycosyl Inositol Phosphorylceramide (GIPC), which is essential components of the outer lipid bilayer, may sensing extracellular salt by directly binding Na^+^. The sodium-bound GIPC interacts with the calcium channel, then activate Ca^2+^ channel to increases cytoplasmic Ca^2+^ concentration (Fig. 3) (Jiang et al. 2019). As a crucial secondary messenger, Ca^2+^ initiates a calcium signaling cascade to modulate plant-wide adaptive responses (Manishankar et al. 2018). The SOS pathway, pivotal for salt tolerance, ensures Na^+^ /K^+^ homeostasis within plant cells. Phosphatidic acid (PA) maintains Na^+^/K^+^ homeostasis by promoting Na^+^ efflux and K^+^ influx, regulating SOS pathway and Arabidopsis K^+^ transporter 1 (AKT1) under salt stress. PA binds to SOS2, a key component of the SOS pathway, and when under salt stress, this promotes the activity and PM localization of SOS2, which activates of the Na^+^/H^+^ reverse transporter SOS1 and to promote Na^+^ efflux. SOS2 also increases the phosphorylation of SOS3-like calcium-binding protein 8 (SCaBP8) in response to salt stress, reducing the inhibition of AKT1 and increasing K^+^ influx (Fig. 3) (Fuglsang and Palmgren 2021; Li et al. 2023). This signaling system allows plants to minimize Na^+^-induced PM damage and maintain integrity (Xiao and Zhou 2023).

**Fig. 4:**
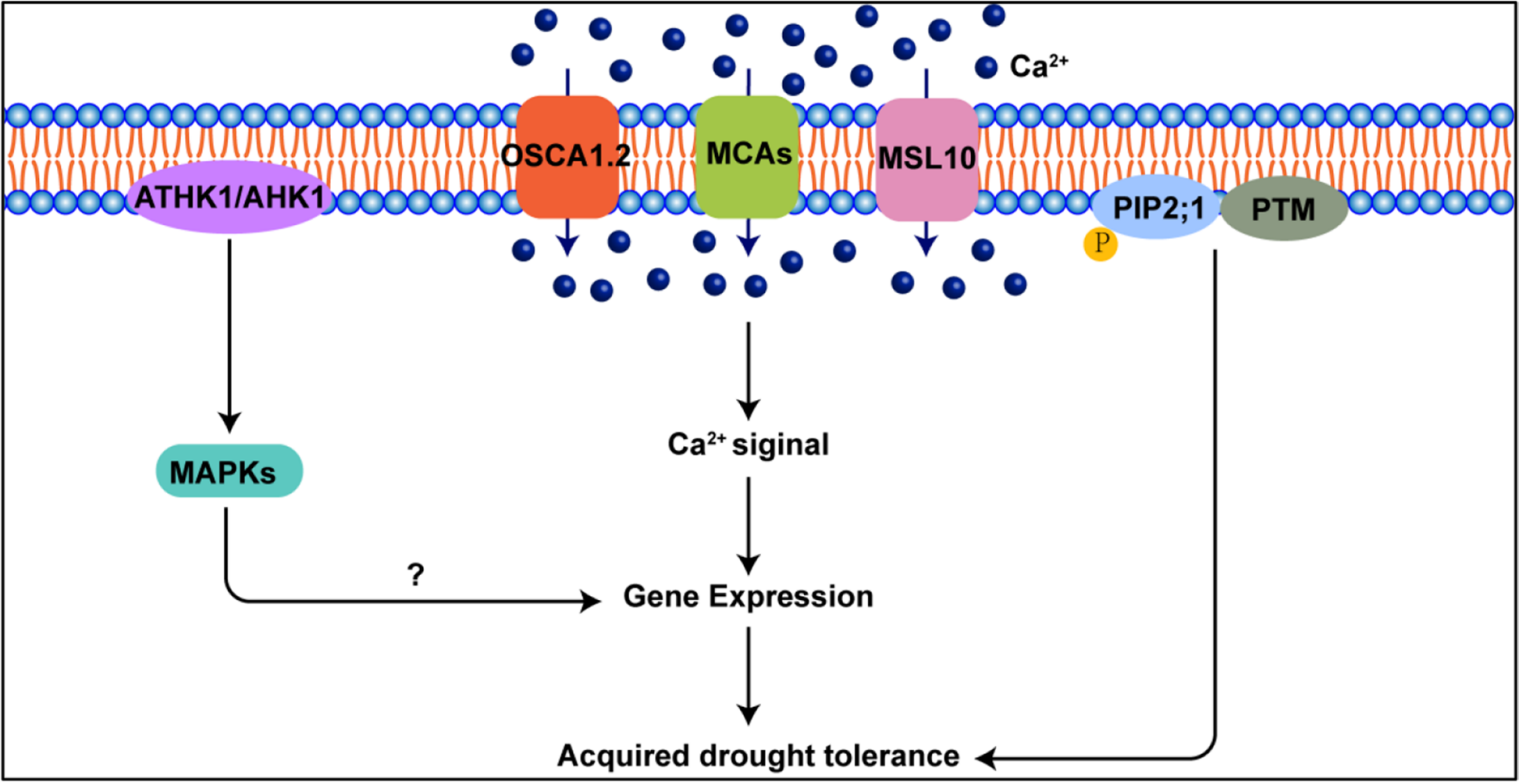
Diagram illustrating the signal transduction pathways that drought stress activates. Under drought conditions, plants experience osmotic stress, which is detected by calcium channels known as MCAs, OSCA1.2 and MSL10. These channels initiate the influx of calcium ions, which then function as secondary messengers to relay signals. ATHK1/AHK1, acting as an osmotic stress signal-sensing protein, subsequently activates MAPKs upon detecting these osmotic stress signals

Although the specific signaling molecules enabling plants to detect water availability changes remain unclear, several studies suggest that changes in external osmolarity directly affect PM mechanics, such as tension and subcellular volumes. Drought leads to osmotic and ionic imbalance, triggering a complex network of osmotic stress signal transduction pathways (Fig. 4) (Mukarram et al. 2021; Gorgues et al. 2022; Kim et al. 2024). Drought leads to osmotic and ionic imbalance, triggering a complex network of osmotic stress signal transduction pathways. Membrane proteins, such as RLKs, histone kinases, and integrin-like proteins, serve as osmotic stress sensors. Arabidopsis histidine kinase 1 (ATHK1/AHK1) at the PM enhances drought tolerance and activates drought response genes, such as the synthesis of proline and sucrose (Fig. 4) (Tran et al. 2007). Although unclear if a MAPK pathway is involved, calcium channels likely perceive osmotic stress in drought responses. The MCA channel, the mechanosensitive (MS) channel of Ca^2+^, is directly triggered by membrane tension to facilitate the flow of Ca^2+^ and the use of Ca^2+^ as a second messenger in signal transduction. (Fig. 4) (Yoshimura et al. 2021). In Arabidopsis, mechanosensitive channel like MscSlike 10 (MSL10) and Reduced hyperosmolality-induced [Ca^2+^]_i_ increase 1.2 (OSCA1.2), detect osmotic stress signals, promoting Ca^2+^ influx to encourage drought-resistant gene expression, maintain PM integrity, and confer drought resistance (Fig. 4) (Yuan et al. 2014; Murthy et al. 2018; Basu et al. 2020; Han et al. 2024). These findings suggest that plants perceive osmotic stress through various proteins on the PM and that precise information is transmitted to all plant tissues through delivering Ca^2+^.

## Conclusion

Plants face numerous abiotic challenges, such as temperature extremes (both high and low), drought, and high salt stress, when exposed to the environment. Although these stresses often do not lead to immediate plant death, they can adversely affect overall plant growth. Several studies have shown that abiotic stress can compromise the integrity of PM. PM repair after damage are crucial for cells maintaining function. However, the underlying mechanisms remain to be further illustrated in plant cells. This review examines the recent findings on how abiotic stress affects PM integrity and how plants respond to such damage. Our goal is to enhance understanding of the relationship between abiotic stress and PM integrity, to explore novel mechanisms by which plants can resist abiotic damage, and to provide a valuable resource for crop breeding research in this review. By shedding light on these interactions, we aim to assist future researchers in developing strategies to improve plant resilience against environmental challenges.

## Data Availability

All data generated or analyzed during this study are included in this published article.
